# A Proteomic View on the Role of Legume Symbiotic Interactions

**DOI:** 10.3389/fpls.2017.01267

**Published:** 2017-07-18

**Authors:** Estíbaliz Larrainzar, Stefanie Wienkoop

**Affiliations:** ^1^Department of Environmental Sciences, Universidad Pública de Navarra Pamplona, Spain; ^2^Department of Ecogenomics and Systems Biology, University of Vienna Vienna, Austria

**Keywords:** proteomics, legume, *Rhizobium*, arbuscular mycorrhizal fungi, pathogen, abiotic stress, drought

## Abstract

Legume plants are key elements in sustainable agriculture and represent a significant source of plant-based protein for humans and animal feed worldwide. One specific feature of the family is the ability to establish nitrogen-fixing symbiosis with *Rhizobium* bacteria. Additionally, like most vascular flowering plants, legumes are able to form a mutualistic endosymbiosis with arbuscular mycorrhizal (AM) fungi. These beneficial associations can enhance the plant resistance to biotic and abiotic stresses. Understanding how symbiotic interactions influence and increase plant stress tolerance are relevant questions toward maintaining crop yield and food safety in the scope of climate change. Proteomics offers numerous tools for the identification of proteins involved in such responses, allowing the study of sub-cellular localization and turnover regulation, as well as the discovery of post-translational modifications (PTMs). The current work reviews the progress made during the last decades in the field of proteomics applied to the study of the legume-*Rhizobium* and -AM symbioses, and highlights their influence on the plant responses to pathogens and abiotic stresses. We further discuss future perspectives and new experimental approaches that are likely to have a significant impact on the field including peptidomics, mass spectrometric imaging, and quantitative proteomics.

## Introduction

The Fabaceae or Leguminosae family, commonly referred to as “legumes,” is the third largest family of flowering plants, second only to cereals in terms of agricultural importance. Some of the most widely studied plants in the family include crops such as soybean (*Glycine max* L. Merr.), common bean (*Phaseolus vulgaris* L.), chickpea (*Cicer arietinum* L.), lentil (*Lens culinaris* Medik.), pea (*Pisum sativum* L.), or alfalfa (*Medicago sativa* L.). However, given the large size and genome complexity of these major crops, the scientific community has focused its efforts in the development of tools and protocols for other legume plants, commonly referred to as model legumes, namely *Medicago truncatula* Gaertn. and *Lotus japonicus* L. For many of these legume species there is genomic sequence information available, which greatly facilitates protein identification using mass spectrometry-based proteomic approaches. Many species within the family are able to establish endosymbiotic relationships with nitrogen-fixing *Rhizobium* bacteria and arbuscular mycorrhizal (AM) fungi. These symbiotic interactions involve complex signal exchanges between both symbionts and an intimate communication to allow the establishment of the bacteria or fungi inside root cells. These interactions are considered mutualistic associations, with *Rhizobium* bacteria providing a source of reduced nitrogen inside specialized root organs named nodules, and AM fungi facilitating the capture of important nutrients for the plant such as phosphorous or sulfur, and even improving the plant responses to biotic and abiotic stress conditions (Ruiz-Lozano et al., 2001; Dimkpa et al., 2009; Pieterse et al., 2014). In order to understand the biological processes governing these symbiotic interactions and their effects on plant fitness, scientists have employed an array of methodologies, ranging from gene expression to proteomic analysis. Proteomics is a powerful tool for the study of subcellular compartmentalization important to understand nodule formation, symbiosome function and to unravel the molecular mechanisms involved in the enhanced stress tolerance of legumes under symbiotic conditions. The current work reviews and summarizes the progress made during the last decades in the field of proteomics applied to the study of legume-*Rhizobium* and -AM symbioses, and their interactions with biotic and abiotic stresses, discussing future perspectives and new experimental approaches.

## Proteomics applied to the study of the legume-*Rhizobium* symbiosis

### Comparative proteomic studies

During the last decades a considerable effort has been made to characterize the diversity of proteins expressed in different tissues under a variety of conditions at the international level. This effort was initiated using 2D-PAGE-based approaches, generating reference maps for various organs in different legume species. Taking *M. truncatula* as an example, Mathesius et al. (2001) were the first to establish a root reference map, which included ~2,500 protein spots, from which 179 were identified. It was remarkable that close to half of them were present as protein isoforms, including key metabolic enzymes such as S-adenosyl-L-methionine synthase, malate dehydrogenase, or ascorbate peroxidase. Gallardo et al. (2003) reported the characterization of the seed proteome during seed filling, with the identification of 84 proteins including proteins belonging to the main storage protein families as well as proteins involved in carbon and sulfur metabolism, among others. Subsequently, works from the Sumner laboratory published a comprehensive analysis of the *M. truncatula* proteome at the different organ level, including cell cultures, with the high-confidence identification of close to 2,000 proteins, the largest proteomic identification reported so far (Watson et al., 2003; Lei et al., 2005).

Focusing on the symbiotic perspective, several comparative proteomics works have been devoted to the analysis of the differential root proteome of a number of legumes when inoculated with their corresponding microbial partners. Depending on the focus of the study, works in the field of symbiosis can be divided in two major groups: (i) studies focused on the characterization of the legume plant proteome, and (ii) works focused on the *Rhizobium* partner (Table 1). In the latter case, a classical comparison is the analysis of the proteome of free-living *Rhizobium* cells vs. their differentiated nitrogen-fixing forms, named bacteroids. This strategy has been applied to identify symbiosis-specific proteins synthesized in the symbionts of the main legume species. Comparison of the proteomic profiles of cultured cells vs. *Sinorhizobium meliloti* nodule bacteroids suggested that nodule bacteria do not express sugar transporters or enzymes involved in the early steps of glycolysis, while containing multiple transporters for nitrogen compounds including amino acids and oligopeptides (Djordjevic et al., 2003; Djordjevic, 2004). The high adaptability of symbiotic bacteria depending on the carbon source was also reported in proteomic works carried out in *Bradyrhizobium japonicum* (Sarma and Emerich, 2005, 2006). Authors reported the unusually low levels in proteins related to fatty acid and nucleic acid metabolism in bacteroids, suggesting that bacteroids and cell cultured bacteria may present differential mechanisms to regulate the levels of ribonucleotides. Subsequent work in *B. japonicum* using more powerful mass spectrometry techniques, however, did not observe such repression in nucleotide metabolism, identifying almost the full set of enzymes involved in *de novo* nucleoside and nucleotide biosynthesis expressed at the gen and/or protein level (Delmotte et al., 2010). Regarding the symbiont of *L. japonicus*, Tatsukami et al. (2013) identified 722 proteins commonly found under the free-living and symbiotic conditions, while 125 proteins were uniquely identified under symbiotic conditions. Interestingly, proteins involved in peptidoglycan biosynthesis and proteins related to the flagellum were uniquely detected under free-living conditions, suggesting that once within the symbiosomes, bacteroids simplify their cell surface by losing their cell wall and motility structures (Tatsukami et al., 2013). Furthermore, the quantitative time-course proteomic analysis of *M. loti* suggested that bacteroids experience nitrogen-deficiency at early stages of nodule development, while at intermediate stages high levels of nitrogenase protein lead to nitrogen-rich conditions in the symbiosome (Nambu et al., 2015).

**Table 1:** Summary of proteomic studies focused on the legume-*Rhizobium* symbiosis.

***Legume* sp**.	***Rhizobium* sp**.	**References**	**Main outcome**
**I. STUDIES FOCUSED ON THE PLANT PARTNER**			
*G. max* roots (wt and *SS2-2* mutant)	*B. japonicum* USDA110	Lim et al., 2010	Protein-mediated suppression of defense-related responses in root cells upon inoculation with symbiotic bacteria.
*G. max* root hairs	*B. japonicum* USDA110	Wan et al., 2005	Induction of phospholipases, phosphoglucomutases, lectins, and an actin isoform in soybean roots upon *B. japonicum* inoculation.
*G. max* nodules (cytosol fraction)	*B. japonicum* USDA110	Oehrle et al., 2008	Proteins related to carbon and nitrogen metabolism, oxygen supply and protection are predominantly found in the cytosol of nodule cells.
*G. max* En-b0-1 roots (supernodulating)	*B. japonicum* MAFF 211342	Salavati et al., 2012	Identification of a correlation between the levels of a peroxidase isoform and nodulation at the protein but not transcript level in soybean nodules.
*L. japonicus* roots and nodules	*M. loti* MAFF30309	Dam et al., 2014	Establishment of 2D-PAGE reference maps of *L. japonicum* roots and nodules.
*M. alba* nodules	*S. meliloti* 1021	Natera et al., 2000	Identification of nearly 100 plant and bacterial proteins in white sweetclover nodules.
*M. truncatula* roots	*S. meliloti* 2011	Bestel-Corre et al., 2002	Two leghemoglobin isoforms and one enolase protein were accumulated in roots upon inoculation with symbiotic bacteria.
*M. truncatula* nodules	*S. meliloti* 2011	Larrainzar et al., 2007	Identification of 377 plant proteins in nodules, mostly related to amino acid metabolism and protein synthesis and degradation.
*M. truncatula* nodules	*S. meliloti* 2011	Larrainzar et al., 2009	Integrative proteomic and metabolomic analysis of the effects of drought stress in the plant and bacteroid fractions of *M. truncatula* nodules.
*M. truncatula* roots and nodules	*S. meliloti* 2011	Larrainzar et al., 2014	Absolute quantification proteomics and gene expression analyses show that sulfur metabolism and ethylene biosynthesis have key roles in the response of nodules and roots subjected to drought stress.
*M. truncatula* shoots and roots	*S. meliloti* 1021	Molesini et al., 2014	Analysis of local and systemic responses of *M. truncatula* roots and shoots upon inoculation.
*M. truncatula* roots (wt and *skl* mutant)	*S. meliloti* 1021	Prayitno et al., 2006	Increased abundance of one ACC oxidase isoform in wild-type roots but not in roots of the supernodulating *skl* mutant upon inoculation.
*M. truncatula* roots and shoots	*S. meliloti* 2011	Staudinger et al., 2012	Detection of salt and drought stress markers and identification of an improved plant response to stress of plants grown under symbiotic conditions when compared to nitrate-fed plants.
*M. truncatula* shoots	*S. medicae* WSM419 and *S. meliloti* 2011	Staudinger et al., 2016	Plants grown under symbiotic conditions present reduced levels of leaf senescence during drought stress independently of the efficiency of the symbiotic *Rhizobium* strain used.
*P. sativum* shoots	*R. leguminosarum* bv. *viciae*	Irar et al., 2014	Identification of variations in protein abundance as part as the local responses of pea nodules grown under split-root conditions and subjected to water stress.
*P. sativum* shoots (soluble and plasma membrane fractions)	*R. leguminosarum* bv. *viciae*	Desalegn et al., 2016	Indications of a positive influence of the symbiotic interaction on the activation of the plant defense responses upon pathogen attack.
*P. sativum* shoots	*R. leguminosarum* bv. *viciae*	Turetschek et al., 2016	Proteomic and metabolomic analyses of two pea cultivars with varying pathogen resistance levels associate tolerance to ethylene biosynthesis and suppression of cell death responses.
*V. unguiculata* roots	*Rhizobium sp*. NGR234	Krause and Broughton, 1992	One of the first proteomic studies analyzing symbiosis-specific proteins potentially involved in root-hair deformation in cowpea.
**II. STUDIES FOCUSED ON THE** ***Rhizobium*** **PARTNER**			
*B. japonicum* USDA110	Bacteroids	Sarma and Emerich, 2005	Abundance of proteins related to nitrogen and carbon metabolism, and transport in soybean nodule bacteroids.
*B. japonicum* USDA110	Free-living cells vs. bacteroids	Sarma and Emerich, 2006	Compared to bacteria under free-living conditions, nodule bacteroids present unusually low levels in proteins related to fatty acid and nucleic acid metabolism.
*B. japonicum* USDA110	Bacteroids	Delmotte et al., 2010	In contrast to previous reports, application of more sensitive LC-MS/MS-based approaches identifies a complete set of proteins related to *de novo* nucleoside and nucleotide biosynthesis in bacteroids.
*Bradyrhizobium sp*. ORS278	Bacteroids (*Aeschynomene indica* root and stem nodules)	Delmotte et al., 2014	Root and stem nodule bacteroids show expression of similar sets of proteins, mostly related to central metabolism. As exceptions, proteins involved in photosynthesis were exclusive found in stem nodules.
*M. loti* MAFF30309	Free-living cells vs. bacteroids	Tatsukami et al., 2013	Differentiated bacteroids do not longer express proteins either involved in peptidoglycan biosynthesis or proteins related to the flagellum.
*M. loti* MAFF30309	Bacteroids	Nambu et al., 2015	Time-course analysis of nodulation suggests that bacteroids experience nitrogen-deficiency at early stages of nodule development.
*S. meliloti* 1021	Free-living cells vs. bacteroids	Djordjevic et al., 2003	Compared to free-living bacteria, nodule bacteroids appear not to require the expression of sugar transporters or enzymes involved in the early steps of glycolysis.
*S. meliloti* 1021	Bacteroids	Djordjevic, 2004	Bacteroids express a specific set of ABC-type transporters involved in the transport of amino acids and inorganic ions.

In the case of plant-oriented studies, it is common to compare differences in the proteome of uninoculated vs. inoculated plants. One of the pioneer works in this line was carried out by Krause and Broughton (1992), reporting 12 symbiosis-specific proteins potentially involved in root-hair deformation in *Vigna unguiculata*, although at the time the lack of genomic sequence information limited protein identification. The availability of expressed sequence tags (ESTs) and, subsequently, genomic sequences of several legume plants have led to greatly improve the number of identified proteins. For instance, the model legume *M. truncatula* has been subjected to detailed proteomic characterization in terms of symbiotic responses. One of the first studies in nodules identified two leghemoglobin isoforms and one enolase protein as some of the proteins that most accumulated in roots upon inoculation with symbiotic bacteria (Bestel-Corre et al., 2002). The protein profiling of the plant fraction of *M. truncatula* root nodules led to the identification of 377 unique proteins, most of them with roles in amino acid metabolism and protein synthesis and degradation (Larrainzar et al., 2007). Proteomics is also a valuable tool for the analysis of local and systemic responses upon inoculation. Through the analysis of the proteomic changes occurring in shoots and roots of inoculated *M. truncatula* plants, 18 proteins were found to accumulate in roots including sucrose synthase 1, a fructose-bisphosphate aldolase, and an alcohol dehydrogenase, while in shoots the content of several proteins involved in defense responses or abiotic stress responses was found to increase (Molesini et al., 2014). Proteomics has been also applied to investigate the effects of the addition of the ethylene precursor aminocyclopropane carboxylic acid (ACC) on nodule development using the supernodulating, ethylene-insensitive mutant *sickle* (Penmetsa and Cook, 1997) during the early stages of the symbiotic interaction (Prayitno et al., 2006). Among other findings, authors observed that *Sinorhizobium* inoculation increased the abundance of one ACC oxidase isoform in wild-type roots but not in *sickle* roots, suggesting that a feedback mechanism regulates the expression this gene. Nevertheless, subsequent work using RNA-seq techniques has shown that at least three genes of the ACC oxidase family are induced in *sickle* upon inoculation (Larrainzar et al., 2015), which highlights the usefulness of combining data at the proteomic and transcriptomic level.

Further characterization of specific metabolic pathways using absolute quantification techniques has been also applied to this model legume, including the detailed analysis of the nitrogen assimilation and ethylene biosynthesis pathways in root nodules (Larrainzar et al., 2009, 2014). Similarly, the symbiotic proteome of soybean, a crop of major economical importance, has been also extensively studied. A time-course proteomic analysis of wild type and the soybean mutant *SS2-2*, which lacks autoregulation of nodulation, has revealed that there is a protein-mediated suppression of defense-related responses in root cells upon inoculation with *Rhizobium* bacteria (Lim et al., 2010). A similar observation was done when comparing the proteomic changes associated to inoculation of soybean plants with differential nodulation capacities (Salavati et al., 2012). In this work, a correlation between the levels of a peroxidase isoform and levels of nodulation was found, although the regulation did not occur at the transcript level. The plant fraction of soybean nodules has been also subjected to proteomic analysis, leading to the identification of 69 proteins mainly related to carbon and nitrogen metabolic activities (Oehrle et al., 2008), similarly to previous observations in *M. truncatula*.

Since root hairs are most frequently the main entry point for *Rhizobium* bacteria, several works have been devoted to identify the proteomic changes occurring in this specialized root cell upon inoculation. This work has the obvious technical limitation that collecting sufficient amount of plant material is challenging and requires a large number of plants per proteomic sample, with estimations of around 1,500 soybean roots and 4,000 soybean seedlings (Wan et al., 2005; Brechenmacher et al., 2009). A time course of the proteomic changes occurring in root hairs revealed that there is a specific induction of phospholipases and phosphoglucomutases, as well as a lectin and an actin isoform upon inoculation (Wan et al., 2005). Under uninoculated conditions, Brechenmacher et al. (2009) combined traditional 2D-PAGE and shotgun methods for the identification of 1,492 proteins present in root hairs, establishing a reference map for future work.

Several recent studies have provided broad insights into the systemic effects the legume-*Rhizobium* symbiotic interaction at the root and, especially, leaf metabolic level (Staudinger et al., 2012, 2016; Desalegn et al., 2016; Turetschek et al., 2016). In all these studies, one of the major response found was related to a significantly induction of the plant translational apparatus and an accumulation of plant proteins involved in stress responses.

### Subcellular proteomics sheds light on protein localization at the symbiotic interface

Proteomic approaches are particularly suited to gain information about protein subcellular localizations. Analyses of enriched fractions or, ideally, purified organelles, or sub-organelle compartments allow the validation of protein compartmentalization and isoform localization data. Furthermore, subcellular fractionation provides valuable information on the specific changes in the proteome of organelles in response to various stresses, allowing for the development of accurate proteomic pathways and networks (Hooper et al., 2014). Legume plants have been also subjected to this type of analysis. Most of work, however, has been done under non-symbiotic conditions. Comprehensive reviews in the field of subcellular proteomics in legumes have been published elsewhere (Lee et al., 2013; Wang and Komatsu, 2016; Yin and Komatsu, 2016). Thus, in the current review we will discuss subcellular proteomic works with a symbiotic focus.

Nodules are complex structures containing a combination of infected and non-infected plant cells. Infected cells are filled with nitrogen-fixing bacteroids arranged in symbiosomes surrounded by a specialized plant membrane named peribacteroid membrane (PBM). The identification of proteins present in this specialized membrane is of key relevance, since it represents the direct interface where nutrient and signal exchange occurs between the legume host plant and *Rhizobium* bacteroids. In order to identify which proteins are localized at the PBM, extensive proteomic work has been carried out in this membrane fraction. Panter et al. (2000) carried out one of the first studies, with the 2D-PAGE analysis of the PBM of soybean nodules. The proteomic characterization of the pea PBM and peribacteroid space, a much more challenging approach, has also been studied (Saalbach et al., 2002). In this work, proteins of the Coatomer-coated vesicles like V-ATPase, BIP, were found in the PBM fraction, supporting the role of the endomembrane system in PBM biogenesis. These studies were followed by more comprehensive LC-MS/MS-based analyses of the PBM in legumes such as *L. japonicus* (Wienkoop and Saalbach, 2003), *M. truncatula* (Catalano et al., 2004), and more recently, soybean (Clarke et al., 2015). Identification of protein components in these membranes has been shown an essential first step for increasing our knowledge on the metabolic exchange processes between plant and bacteroid. For instance, the proteomic analysis of the PBM in *L. japonicus* led to the identification of a symbiosis-specific sulfate transporter 1 (SST1) and around 80 other abundant membrane or membrane-associated proteins such as the hypersensitive response protein and remorin (SYMREM1; Wienkoop and Saalbach, 2003). Both SST1 and SYMREM1 have subsequently been characterized in detail and shown to be relevant for nodule development and functioning (Krusell et al., 2005; Lefebvre et al., 2010; Toth et al., 2012; Domonkos et al., 2013; Kalloniati et al., 2015).

### Post-translational modifications fine-tune symbiotic events

One of the strengths of mass spectrometry-based proteomic approaches is that it allows the identification of post-translational modification (PTM) sites in proteins, something that cannot be accurately predicted with genomic information alone. PTMs have a huge impact on plant signaling and metabolism, contributing to the regulation of protein activity, stability/degradation, interactions, and ultimately gene expression. In recent years, the number of studies focused on the identification of PTMs in plants has considerable grown (for an extensive review, see Friso and van Wijk, 2015). Particular interest has received the large-scale identification of phosphoproteins at early stages of the legume-*Rhizobium* symbiotic interaction. The reason behind this interest is that a number of protein kinases have been shown essential for rhizobial infection and/or nodule development. These include LysM-domain-containing receptor kinases, implicated in the binding of Nod factors, as well as other membrane receptor-like kinases and calcium/cadmodulin-dependent protein kinases (Antolín-Llovera et al., 2014). Analysis of the phosphoproteome at early symbiotic stages has been carried out in *L. japonicus* roots (Serna-Sanz et al., 2011), soybean root hairs (Nguyen et al., 2012), and *M. truncatula* roots both applying discovery proteomics (Rose et al., 2012) and targeted approaches (van Ness et al., 2016). These studies are important first steps toward understanding how Nod-factor signaling is transmitted from the plasma membrane and decoded to activate the developmental reprogramming root cells undergo to allow nodule formation. Previous studies identified several phosphoproteins present in mature nitrogen-fixing nodules, including several phosphopeptides in sucrose synthase 1 and alkaline invertase, the main sucrose-degrading enzymes in this specialized organ (Wienkoop et al., 2008). Similarly, large-scale analyses of *M. truncatula* roots (Grimsrud et al., 2010) or during nodulation (Marx et al., 2016) have allowed the identification of a repertoire of *in vivo* phosphorylated peptides and phosphorylation motifs, which can be queried online (http://www.phospho.medicago.wisc.edu and http://compendium.medicago.wisc.edu, respectively).

Ubiquitination is another PTM that plays important roles for rhizobial infection and nodule organogenesis in legumes. For instance, proteins such as the receptor kinase *M. truncatula* LYK3 has been found ubiquitinated *in vitro* by the E3 ubiquitin ligase PUB1 (Mbengue et al., 2010) and a deubiquitinating enzyme named AMSH1 has been shown to be required for the establishment of an effective symbiosis in *L. japonicus* (Małolepszy et al., 2015). However, to our knowledge, the large-scale identification of ubiquitinated proteins under symbiotic conditions has not been carried out to date.

The role of the signaling molecule nitric oxide and its associated protein modifications, nitrosylation and nitration, has also drawn considerable attention in legume studies. At the level of root nodules, two proteins have been identified as targets of Tyr nitration: the ammonium-assimilating enzyme glutamine synthetase (GS; Melo et al., 2011; Blanquet et al., 2015) and the hemeprotein leghemoglobin (Sainz et al., 2015). In the latter work it was found that leghemoglobin not only plays an important role as an O_2_ transporter but may also act as sink of toxic peroxynitrite and thus be part of a protective mechanism in symbiosis.

Other PTM studies in the field include the identification of sulfenylated proteins in the *M. truncatula-S. meliloti* symbiosis (Oger et al., 2012). Interestingly, both in the plant and bacterial partners a large proportion of the identified proteins were related to the glycolytic pathway, tricarboxylic acid cycle and amino-acid metabolism, including one of the nodule-enhanced sucrose synthase isoforms. Additionally, a cytosolic isoform of glutamine synthetase has been found as a target of sulfoxidation, although this modification did not alter the activity of the enzyme (Matamoros et al., 2013).

## Proteomic studies on the legume-AM fungi symbiosis

The plant-AM fungi (Glomeromycota) is the most extensively observed association with roots of land plants (>80%; Schüβler et al., 2001). The symbiotic interaction includes formation of appressoria on the root surface, the entrance into the root epidermis, proliferation within the cortical parenchyma and the formation of arbuscule structures (Giovannetti et al., 1996). These hyphal branches are surrounded by a plant-derived plasma membrane, called periarbuscular membrane (Alexander et al., 1989; Harrison, 1999). Similarly to the PBM, this membrane is the actual site of the plant-microbe interaction. AM symbiosis is described as a bilateral nutritional beneficial association whereby AM fungi supply plants primarily with phosphorus and also nitrogen, while plants provide corporate fungi with carbohydrates (Harrison, 1999). At the gene level, the induction of phosphate transporter genes during AM symbiosis has been reported in several plant species (Rausch et al., 2001; Harrison et al., 2002; Paszkowski et al., 2002). By analyzing the protein profiles of the periarbuscular membrane, the localization of such transporters can be confirmed, as demonstrated in the model legume *M. truncatula* (Harrison et al., 2002). Further proteomic works in this legume have identified changes in the levels of H1-ATPase and a predicted glycosylphosphatidylinositol-anchored blue copper-binding protein in response to AM-association (Gianinazzi-Pearson et al., 2000; Bestel-Corre et al., 2004; Valot et al., 2006). The high H1-ATPase activity was described to support the existence of an active nutrient transport between the partners (Ferrol et al., 2002).

Recently, Abdallah et al. (2014) examined the profile of the *M. truncatula* root membrane proteome after microsomal enrichment. The most abundant organelle components that were retrieved encompassed the plastid, the nucleus and the plasma membrane. Comparing AM-colonized vs. non-mycorrhized plants, they found a lysine/histidine transporter, as well as a differential abundance of about 100 other proteins upon mycorrhization, including known sulfate transporters, the above described H1-ATPase and blue copper protein. Comparison between mutants with contrasting AM-colonization genotypes showed differences at the level of appressorium-responsive proteins (Amiour et al., 2006). Another proteomic study on *M. truncatula* roots colonized with two different *Glomus* species identified a conserved plant responses to mycorrhizal colonization, which include proteins related to redox homeostasis, carbon metabolism, and energy generation (Recorbet et al., 2010). In this regard, a closer look into the root plastid proteome revealed that arbuscule development was potentially slowing down the hosts anabolic reactions such as N assimilation, fatty acid biosynthesis, glycolysis, and pentose phosphate pathway (Daher et al., 2016). Authors also proposed that the reduced C and N assimilation was concurrent with the reallocation of other molecules, possibly to be stored as N-rich compounds. In addition and in accordance with investigations of the leaf proteome of *P. sativum* (Desalegn et al., 2016), they also found an AM symbiosis-induced oxidative stress signature.

All in all, a strong influence of the AM symbiosis on the plant metabolism has been evidenced by these proteomic studies, a response that may be comparable to the previously described systemic resistance induced by rhizosphere bacteria (van Loon et al., 1998).

## Proteomics as a tool to identify stress responses in legumes under symbiotic conditions

### Legume-microbe interactions and abiotic stress alleviation

Although a number of proteomic studies have been published on the response of legumes to abiotic stresses, in most studies, however, plants under study have not been grown under symbiotic conditions. For instance, analyses of peanut (*Arachis hypogaea* L.) varieties with contrasting tolerance to water deficit have shown a relation between plant tolerance to the stress and the abundance of proteins involved in stress signaling and wax biosynthesis in leaves (Kottapalli et al., 2009). A similar approach was taken by Katam et al. (2016) who identified proteins related to nitrogen metabolism, defense and cellular biogenesis exclusively in the tolerant peanut cultivar. Abiotic stress responses in a major legume crop like soybean have been reviewed elsewhere (Hossain et al., 2012, 2013; Hossain and Komatsu, 2014; Komatsu et al., 2015). Here we will focus on the proteomic works analyzing the effects of abiotic stress of legumes grown under symbiotic conditions.

Regarding the legume-*Rhizobium* symbiosis, the effects of drought stress on the *M. truncatula* nodule proteome have been characterized in most detailed. Proteomic analysis of the plant protein fraction of drought-stressed nodules showed a general decline in enzymes involved in symbiotic nitrogen fixation and N assimilation (Larrainzar et al., 2007). In contrast, the microsymbiont showed an accumulation of chaperonins and heat-shock proteins, along with enzymes involved in energy metabolism. In a later study, the application of an integrative proteomic and metabolic approach to *M. truncatula* nodules allowed the identification of the main effects of drought followed by a recovery treatment (Larrainzar et al., 2009). Both works highlighted the role of enzymes related to sulfur assimilation in nodules, with the identification of a specific, nodule-enhanced methionine synthase isoform responding to water deficit conditions. Thus, in a subsequent work, Larrainzar et al. (2014) analyzed in detail the involvement of sulfur metabolism in the nodule response to drought. Gene expression and absolute quantification of proteins in the methionine and ethylene biosynthesis pathways were found strongly down-regulated during drought, demonstrating the contribution of nodule sulfur metabolism and the phytohormone ethylene for nodule functioning under water deficit conditions. Using a split-root system and proteomics, local inhibition of nitrogen fixation and nodule metabolism was evidenced in *M. truncatula* and soybean plants partially exposed to drought stress (Gil-Quintana et al., 2013). More recently, the drought response of two legume species was compared, finding a similar molecular response to drought but a higher drought tolerance in the tropical legume (Gil-Quintana et al., 2015). In this study, protein isoforms that share same function and location could be identified across species.

Induced systemic resistance and the positive effect of microbes in the plant immune system has received considerable in recent years (van Wees et al., 2008). In this line, the effect of *Rhizobium* in priming plant metabolism in response to salt and drought stress has been analyzed in *M. truncatula* using a combination of proteomic and metabolomic techniques (Staudinger et al., 2012, 2016). These works highlighted the involvement of another group of nodule proteins in the plant response to drought; the family of lipoxygenases, enzymes related to lipid metabolism and jasmonate biosynthesis. Additionally, authors found an influence of the *Rhizobium* symbiosis on the abundance levels of stress responsive proteins prior application of the stress treatment. In subsequent works, plants grown under symbiotic conditions were found to present reduced levels of leaf senescence under drought stress, regardless the efficiency of the *Rhizobium* strain used (Staudinger et al., 2016). Authors concluded that, besides increased jasmonate biosynthesis and potassium ion concentrations, allocation of reserves to osmolytes and a shift in carbon partitioning from starch to sugar are key responses for the observed symbiont-induced stay-green effect.

The plant-AM symbiosis has been shown to have a positive impact on the plant nutritional status, thus, improving growth and crop productivity. In legumes, an overall enhanced tolerance to abiotic stress has been also reported (Ruiz-Lozano et al., 2001). However, specifically in the field of proteomics applied to the legume-AM symbiosis, works describing this positive effect are scarce. One of the few examples are the analyses by Aloui and colleagues focused on the changes in the proteome of *M. truncatula* roots (Aloui et al., 2009) and shoots (Aloui et al., 2011) when colonized with the AM fungus *G. intraradices* in cadmium (Cd)-free and Cd-contaminated substrates. Authors identified nine out of 15 proteins changing under Cd treatment in non-mycorrhizal roots that were not observed in Cd-treated, colonized roots. In shoots Cd induced the accumulation of proteins related to photosynthesis in plants under symbiotic conditions. As a conclusion, when exposed to Cd, symbiotically grown plants partially escaped metal toxicity through a concerted increase in shoot biomass and thanks to allocation plasticity strategies compared to non-symbiotic plants.

**Figure 1:**
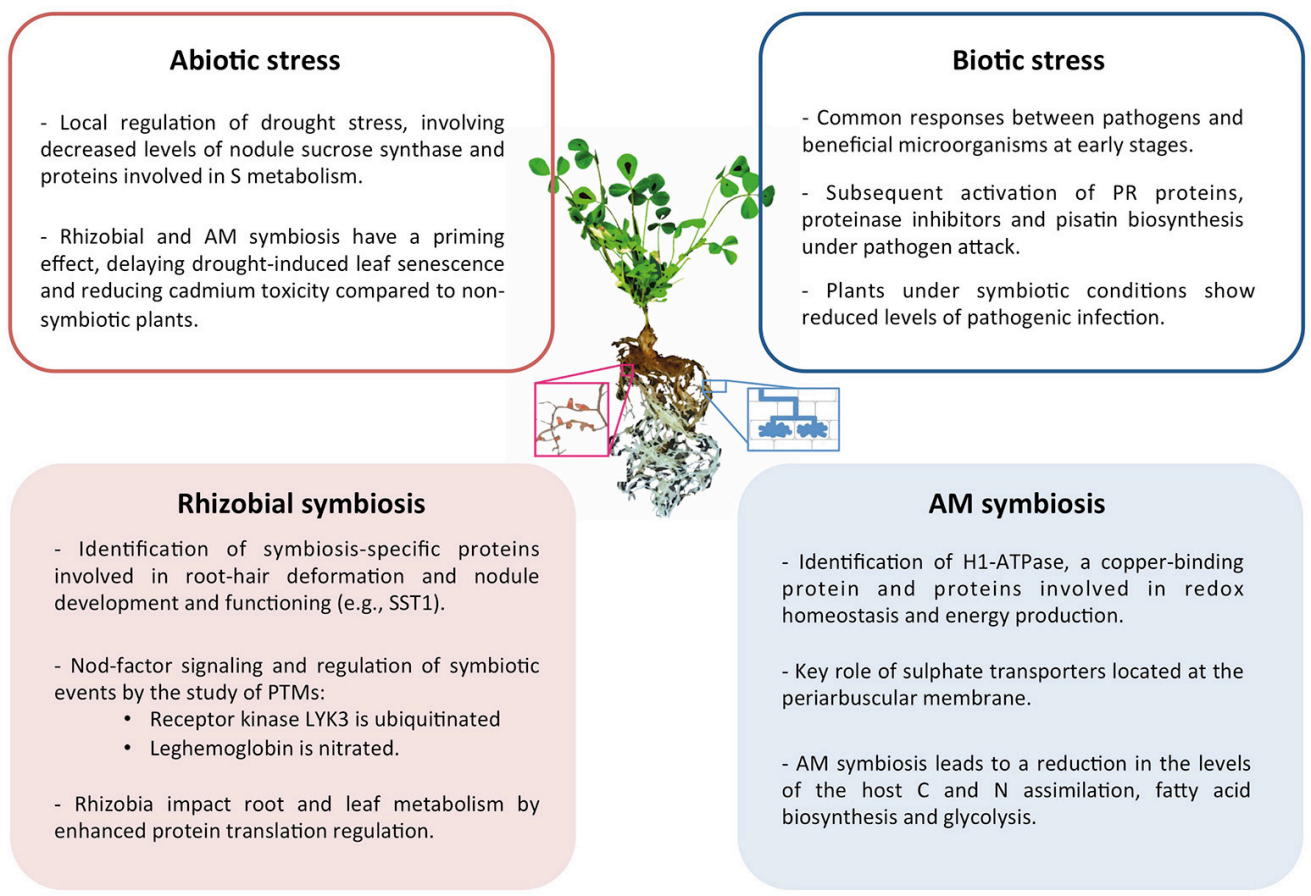
Summary of the main conclusions drawn from proteomic studies of symbiotic legume plants and their interactions under abiotic and biotic stress conditions. Center image represents a *M. truncatula* plant and a magnified image of nodulated roots (left) and schematic representation of cells containing AM fungi (right).

Collectively, in the last decade proteomics research has provided a significant contribution to the understanding of the legume symbiotic interactions in response to biotic and abiotic stress. A summary of the main conclusions drawn can be found in Figure 1.

### Biotic stress responses and plant defense under symbiotic conditions

Symbiotic interactions and responses to a pathogen attack share some features especially at the early stage of the interaction. Legume hosts initially recognize their symbiotic partners as potential threats, activating plant defense responses that are subsequently down-regulated at later stages (Zamioudis and Pieterse, 2012). Since plants under symbiotic conditions appear to show an improved tolerance to abiotic stresses, it could be hypothesized that a similar pattern would be observed when challenged by pathogens. In terms of proteomic analysis, few are the works that have analyzed this question and most studies have been carried out on legumes grown under non-symbiotic conditions. One of the few works on the topic analyzed the proteomic changes occurring during the tripartite interactions with *S. meliloti*, mycorrhizal fungi, and the pathogenic oomycota *Aphanomyces euteiches* in *M. truncatula* roots (Schenkluhn et al., 2010). Among the proteins detected after inoculation either with *G. intraradices* and/or *S. meliloti* the highest increase in relative content was observed for a calmodulin-2, which could be related to the activation of Nod- and Myc-factor signaling cascades. In agreement with previous studies, authors observed the activation of proteins involved in antioxidant defense and/or scavenging of reactive oxygen species. At later stages the pathogen induced the expression of a different set of proteins including pathogen response (PR) proteins, Kunitz-type proteinase inhibitors, a lectin, and proteins related to primary carbohydrate metabolism. Interestingly, the induction of these pathogen responses occurred to a lesser extent in plants under mixed infections (i.e., symbiotic plants infected with *A. euteiches*), which indeed reinforces the idea of the beneficial effects of symbiosis toward pathogenic infections. A similar observation was made when analyzing the leaf proteome and metabolome of pea plants after the co-inoculation with *R. leguminosarum* and AM fungi, or either of those combined with the pathogenic fungi *Didymella pinodes* (Desalegn et al., 2016). The *Rhizobium* symbiosis considerably increased the levels of proteins involved in the pisatin pathway upon pathogen attack compared to mycorrhized, co-inoculated plants or plants under non-symbiotic conditions. Hence, it was concluded that proteins and pathways involved in symbiotic interactions might indirectly be linked to specific pathogen-response pathways. However, the regulatory link between the different molecular responses induced by symbionts and pathogens remains unknown. In fact, when in a subsequent work the effect of the symbionts and pathogen disease levels were compared on two pea cultivars with differential susceptibility, the influence of the microsymbiont was found superimposed by genotypic resistance traits (Turetschek et al., 2016).

## Integrative plant proteomics and legume/symbiont databases

The storage and public availability of large proteomic data resources is of great interest for the wider distribution of proteomic data in the scientific community. Several databases give either access to proteome sequence information of fully sequenced genomes and their functional annotation such as the Universal Protein Resource (UniProt, http://www.uniprot.org) or even of mass spectrometry spectral identification like ProMEX (Hummel et al., 2007; Wienkoop et al., 2012). Nevertheless, to date genomic databases for most plant model species, including legumes, lack proteome subcellular localization annotation (Hooper et al., 2016). One of the best-characterized subcellular proteome is that of the non-legume model plant *A. thaliana*, which is stored in the SUBA3 database (Tanz et al., 2013). Recently, subcellular localization data based on gene co-expression have been extended for agriculturally relevant plants including soybean (Obayashi et al., 2014). An extension of this database integrating proteomic data of those species has been also made publically available (Hooper et al., 2016).

Although a summary of the main databases specific for legume plants has been published elsewhere (González et al., 2015; Ramalingam et al., 2015), the current section reviews some additional databases that were not included above. The platform for integrative legume biology (LEGOO; https://www.legoo.org) offers a tool for the automatic annotation of several legume proteomes including these of *M. truncatula, G. max* and *L. japonicus*. One of the strengths of this database is that it allows for the rapid identification of orthologous genes/proteins across different legume species, simplifying the task of tracking down proteins with multiple IDs. Recently, Lotus Base an integral information portal providing genomic, proteomic, and expression resources for the model legume *L. japonicus* has been also made available (https://lotus.au.dk; Mun et al., 2016). Regarding data on post-translational modification, Schulze et al. (2015) have summarized the online databases available in the field of phosphoproteomics for a range of plant species, including the model legume *M. truncatula*.

In terms of databases focused on the microsymbionts, one useful platform is the RhizoGATE (http://www.cebitec.uni-bielefeld.de/CeBiTec/rhizogate; Becker et al., 2009), which collects several databases containing genomic and transcriptomic information of a number of *Rhizobium* species. Similarly, the RhizoBase (http://genome.microbedb.jp/rhizobase; Fujisawa et al., 2014) is a manually-curated genome annotation database containing genome data for *Rhizobium*, including the option of downloading data on several microsymbiont proteomes. However, to our knowledge, the only database containing proteomic spectral and experimental metadata on *Rhizobium* is ProMEX, including spectral peptide information on *S. meliloti* (955 entries) and *B. japonicum* (157 entries).

One major challenge of proteomic data is that the vast amount of information generated is difficult to present in a convenient, accessible way, thus mostly ending up in huge lists of metadata that are difficult to access for further work. Additionally, transfer of knowledge from one organism to another is very restricted; even if protein functions can be estimated based on sequence similarity, their localization and specific responses to stress varies depending on the plant species. One example of such comparative approach can be found in Gil-Quintana et al. (2015), who compared the *M. truncatula* and soybean root nodule proteome in response to drought. Authors found that the nodule proteome in response to stress in grain and forage legumes was very similar, suggesting that proteome research conducted on the model legume might be extended to other economically relevant crop legumes.

Taken together, there are several legume-specific proteomic databases available. The development of a platform gathering and allowing the analysis of this proteomic information in legumes, similarly to the MASCP gator for the visualization and extraction of proteomic information in *A. thaliana* (Joshi et al., 2011), might be a future goal in the legume-symbiosis community.

## Conclusion and future perspectives

This work reviews some of the most important outcomes of proteomic analysis of the legume-*Rhizobium* and -AM symbiosis and their interactions with abiotic and biotic stresses. It has become evident that symbiotic interactions have a significant, positive impact on the plant fitness levels, improving their tolerance to stress conditions and pathogen attack. Although, supplementation based on nitrogen fertilizers under optimal conditions may still lead to higher crop yield in global terms, the positive influence of the microsymbiont should be taken into consideration for future breeding programmes, particularly now under the predicted climatic change scenarios.

During the last decade, improvements in terms of speed and accuracy of mass spectrometric instruments has led to about one order of magnitude increase in the amount of high-throughput proteomic data obtained, with the identification of >1,000 different proteins per experiment. However, these improvements have not yet been fully exploited in the field of the legume-microbial symbiosis. There is, for instance, growing interest in extending the work on endogenous plant peptides described to act as signaling molecules and shown to be involved in plant-microbe communication (van de Velde et al., 2010). Hence, application of peptidomic techniques may significantly contribute to unravel the plant control mechanisms for both beneficial and pathogenic interactions in the root microbiome. One example of potential applications is the use of MALDI mass spectrometric imaging, a technique that allows for the visualization of spatial protein distributions, recently applied to analyze changes in the levels of endogenous peptides and proteins at different developmental stages in *M. truncatula* (Ye et al., 2013; Gemperline et al., 2016). Similarly, other relatively novel proteomic techniques are slowly being introduced in the field, including, isotopic labeling strategies for protein turnover (Lyon et al., 2014, 2016), or absolute quantification studies (Lehmann et al., 2008; Larrainzar et al., 2009). Combinations of spatial and temporal experiments are rare and there is a need of performing phenotyping of protein abundance at the organelle level to improve our understanding of the legume symbiotic interactions. In this regard, it would be of great interest to connecting protein localization with organelle abundance profiling (Parsons and Heazlewood, 2015). This can be done through the combination of (i) unbiased approachs using e.g., spectral count or LFQs (Maxquant; Hoehenwarter and Wienkoop, 2010; Wienkoop, 2013a) for all proteins belonging to a target organelle (provided that subcellular localization is known) and (ii) selective reaction monitoring (Wienkoop, 2013b; Recuenco-Munoz et al., 2015) of proteotypic peptides selected from abundant and organelle-specific proteins. Also, non-aqueous fractionation, a technique that has been demonstrated to be useful for the integrative subcellular analysis of metabolites and proteins (Arrivault et al., 2014), has thus far not been applied for legume symbiosis research.

Taking into account the large number of proteins involved in response to abiotic and biotic stresses that have been identified, it is now necessary to integrate and compile this information in an accessible way so that this proteomic data and the numerous potential markers identified are analyzed in terms of their contribution to symbiotic and pathogen signaling, nodule formation and nitrogen-fixation efficiency, as well as in improved stress tolerance. The growing application of CRISPR/Cas9 technology and the availability of mutant lines for several model legumes will most likely contribute to this end. Interactions between plant genomic and proteomic specialists and integration of their techniques are key aspects that need to be strengthened in the future.

## Author contributions

EL and SW performed the literature review, analysis, drew conclusions, and wrote the article.

### Conflict of interest statement

The authors declare that the research was conducted in the absence of any commercial or financial relationships that could be construed as a potential conflict of interest.

## Abbreviations

2D-PAGE: two-dimensional polyacrylamide gel electrophoresis
AM: arbuscular mycorrhizal
CRISPR: clustered regularly interspaced short palindromic repeats
LC-MS/MS: liquid chromatography coupled to tandem mass spectrometry
MALDI-TOF: matrix-assisted laser desorption/ionization-time of flight mass spectrometry
PBM: peribacteroid membrane
PR: pathogen response
PTM: post-translational modification
SST1: symbiosis-specific sulfate transporter 1.
